# Extensive LDL-cholesterol lowering by PCSK9 inhibitor on the risk of venous thrombosis

**DOI:** 10.1093/ehjcvp/pvae077

**Published:** 2024-11-08

**Authors:** Shinya Goto, Shinichi Goto

**Affiliations:** Department of Medicine (Cardiology) Tokai University School of Medicine, 143 Shimokasuya, Isehara 259-1193, Japan; Department of Medicine (General Internal Medicine), Tokai University School of Medicine, 143 Shimokasuya, Isehara 259-1193, Japan

**This editorial refers to ‘Role of PCSK9 inhibitor in venous thromoembolism: current evidece and unmet needs’, by Zuin et al. doi: 10.1093/ehjcvp/pvae076.**

Proprotein convertase subtilisin/kexin type 9 inhibitors (PCSK9i) have recently emerged as a new therapeutic option to reduce low-density lipoprotein cholesterol (LDL -C) extensively. Clinical trials demonstrated the additional effect of PCSK9i in reducing cardiovascular (CV) events compared to commonly used statins alone.^1^ The main mechanism of lowering CV events depend on the plaque stability achieved by LDL -C lowering.^2^ Indeed, the vast majority of acute coronary syndrome (ACS) occurred as thrombosis initiated at the sites of vessel damage caused by lipid rich plaque disruption.^3^ LDL -C lowering might also influence the thrombogenicity through modification of coagulation cascade or platelet function.^4^ However, the antithrombotic effects mediated by LDL -C lowering has not been extensively investigated so far.

In this issue of EHJ-CVP, Zuin, *et al.* summarized an interesting overview regarding the potential benefits of PCSK9i for the prevention and treatment of venous thromboembolism (VTE).^5^ Unlike ACS, VTE is not hugely influence by plaque rupture. Anticoagulation therapy is the standard of care for prevention and treatment of VTE. Recent global registry revealed the high residual risks of VTE recurrence of 5.4% (95% CI: 4.9–5.9)/12 month despite treatment availability.^6^ Widespread use of anticoagulants such as recently developed direct oral anticoagulants should reduce the risk of VTE. However, the risk of serious bleeding complication limits the use of anticoagulants, especially in patients with high risks of bleeding.^7^

LDL -C lowering might be helpful in reducing the risk of VTE without increasing the risk of serious bleeding complications. Indeed, a previous randomized clinical trial revealed lower risk of VTE in patients treated by rosuvastatin.^8^ Despite the presence of supportive clinical evidences,^9^ statin did not get a label for indication in VTE prevention and treatments. Now, PCSK9i, a stronger LDL -C lowering agent became available. The authors reminded us that we can expect an approximately 31% risk reduction of VTE (Hazard Ratio (HR): 0.69 0.53–0.90 95% CI) by the use of PCSK9i.^10^ As beautifully summarized by the authors, PCSK9is could reduce the risk of VTE recurrence by their lipid-related or non-lipid-related effects such as inhibition of platelet activation and procoagulant activities by inhibiting signal transduction by the lipid raft.^11^

The potential benefit in prevention of VTE by PCSK9i summarized by Zuin is important. The risk of VTE is likely to be decreased by LDL -C lowering. PCSK9i is a relatively novel class of drugs compared to statin. Off-label use of PCSK9i is not recommended. Accordingly, we expect someone (likely to be companies developing PCSK9i) to conduct phase III clinical trials to get new indication of VTE prevention in the future.

Platelets and leukocyte circulate in whole body are the main blood cells mediating thrombogenicities and inflammation. They stay mostly in microvessels, including capillary, microarteries, and microveins. Both platelets and leukocytes interact with endothelial cells in the whole body.^12^ Both antithrombotic and anti-inflammatory functions of endothelial cells are impaired by exposure to various risk factors such as diabetes, high blood pressure, or LDL -C. The risk of VTE increases in the presence of blood containing partially activated platelets/leukocytes (upper panel) *Fig. 1*. Strong LDL -C lowering agents, such as PCSK9i, could decrease the risk of VTE by maintaining the healthy state of vascular endothelial cells in whole body (lower panel).

**Figure 1:**
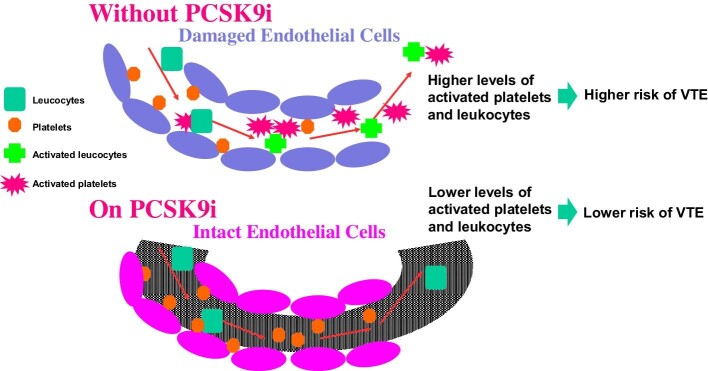
Potential importance of LDL -C lowering for prevention of venous thromboembolism.

## Notes

**Conflict of interest:** The author Shinya Goto declare that he is the member of executive committee for CORALreef Outcomes (TIMI 77) trial for the clinical development of orally available PCSK9 inhibitor sponsored by Merck. Shinya Goto also declare that he is a Member of the Executive Committee for the clinical development of anti-XI agents sponsored by Jansen and Bristol Myer Squibb. Shinya Goto is the Steering Committee Member for the clinical development of other lipid modifying agent namely Olpasiran sponsored by Amgen, anti-XI agents sponsored by Anthos Therapeutics. Shinya Goto is an associate editor for circulation. The author Shinichi Goto has nothing to declare.

## Data availability

There are no new data associated with this article.
